# Genome Sequence of the Tick-Borne Pathogen *Rickettsia raoultii*

**DOI:** 10.1128/genomeA.00157-16

**Published:** 2016-04-21

**Authors:** Khalid El Karkouri, Oleg Mediannikov, Catherine Robert, Didier Raoult, Pierre-Edouards Fournier

**Affiliations:** Unité de Recherche en Maladies Infectieuses et Tropicales Emergentes (URMITE), UM63, CNRS7278, IRD198, INSERM U1095, Institut Hospitalo-Universitaire Méditerranée-Infection, Aix-Marseille Université, Faculté de Médecine, Marseille, France

## Abstract

*Rickettsia raoultii* is a tick-associated spotted fever group (SFG) organism, causing scalp eschar and neck lymphadenopathy after tick bite (SENLAT) in humans. We report here the genome sequence of *R. raoultii* strain Khabarovsk^T^ (CSUR R3^T^, ATCC VR-1596^T^), which was isolated from a *Dermacentor silvarum* tick collected in Russia.

## GENOME ANNOUNCEMENT

*Rickettsia* species are obligate intracellular alphaproteobacteria that can infect humans or animals, mostly through arthropod bites, and they can cause a range of mild to fatal diseases, such as spotted fevers and murine or epidemic typhus. The spotted fever group (SFG) species *Rickettsia raoultii* can cause scalp eschar and neck lymphadenopathy after tick bite (SENLAT) in humans (1). Originally detected in *Dermacentor nuttalli* and *Rhipicephalus pumilio* ticks, *R. raoultii* was then detected in a *Dermacentor silvarum* tick (2, 3) prior to being identified mainly in *Dermacentor* species ticks worldwide (1). Here, we briefly describe the genome sequence of *R. raoultii* strain Khabarovsk^T^ isolated from *D. silvarum* ticks collected in Russia in 2005 (3) and inferred the phylogenomic position of the species.

The genome was sequenced using the GS20 and GS FLX Titanium platforms (100-bp single ends, 3-kb paired-end reads; Roche, CT), and the Genome Analyzer II technology (200-bp paired-end reads; Illumina, USA). The *de novo* read assemblies and mapping were performed using the Newbler and CLC Genomics softwares. Most gaps between contigs were closed using specific PCRs. The protein-coding genes were predicted using AMIGene (4). Assignment of protein functions was performed by searching against RickBase and GenBank using BLASTp (5). Gene classification into functional categories was processed using COGsoft (6). Ribosomal RNAs, tRNAs, and other RNAs were identified using RNAmmer, tRNAscan-SE, and BLASTn (7–9). Membrane and secreted proteins were predicted using TMHMM (http://www.cbs.dtu.dk/services/TMHMM/), SignalP, and SecretomeP (10, 11). Orthologous genes between 29 *Rickettsia* species were identified using COGsoft, with a coverage of ≥80% and an *E* value ≤10^-10^ (6). Multiple sequence alignment of 330 concatenated core genes was carried out using MAFFT (12). Phylogenomic inference was performed using maximum likelihood with GAMMA and JTT models with 100 bootstrap (BP) replicates.

*R. raoultii* exhibited one chromosome of 1,344,605 bp (containing one gap), 1,334 genes, and a G+C content of 32.5%. It encoded 11.7, 2.8, and 1.1% of membrane, exported, and alternative secreted proteins, respectively. Three rRNAs (16S, 23S, and 5S rRNA), one transfer-messenger RNA (tmRNA), two misc_RNAs, and 33 tRNAs were identified. Compared to the more virulent *Rickettsia slovaca*, *R. raoultii* had more genes involved in replication, recombination, and repair (30 versus 7), translation (10 versus 6), transduction (16 versus 4), and transport and metabolism (39 versus 21). The phylogenomic analysis of 29 *Rickettsia* species identified a reliable cluster (bootstrap, 100%) grouping *R. raoultii*, *Rickettsia aeschlimannii*, *Rickettsia massiliae*, and *Rickettsia rhipicephali* together.

Moreover, the *R. raoultii* genome contained the three plasmids pRra1, pRra2, and pRra3, having sizes ranging from 20,840 to 83,219 bp. These plasmids were closely related to *R. massiliae* pRma, *Rickettsia helvetica* pRhe, and *Rickettsia buchneri* pReis3, respectively (13). The pRra2 plasmid harbored a conjugative TRA cluster. However, the presence of a cluster of 6 genes in the *R. raoultii* chromosome that matched *Rickettsia peacockii* pRpe plasmid (amino acid identity, 95 to 98%) and *Pseudomonas aeruginosa* chromosomes (amino acid identity, 62 to 82%) suggests that it may be horizontally transmitted and/or *R. raoultii* might have harbored a fourth plasmid that was disrupted during the reductive evolution.

### Nucleotide sequence accession numbers.

The whole-genome shotgun sequences were deposited in GenBank under accession numbers CP010969, CP010970, CP010971, and CP010972.
